# HMGB1 is a Potential Mediator of Astrocytic TLR4 Signaling Activation following Acute and Chronic Focal Cerebral Ischemia

**DOI:** 10.1155/2020/3929438

**Published:** 2020-02-20

**Authors:** Bolanle M. Famakin, Orest Tsymbalyuk, Natalia Tsymbalyuk, Svetlana Ivanova, Seung Kyoon Woo, Min Seong Kwon, Volodymyr Gerzanich, J. Marc Simard

**Affiliations:** ^1^Department of Neurology, University of Wisconsin, 1685 Highland Ave, Madison, WI 53705, USA; ^2^Department of Neurology, University of Maryland School of Medicine, 110 S. Paca St., Baltimore, MD 21201, USA; ^3^Department of Neurosurgery, University of Maryland School of Medicine, 22 S. Greene St., Suite S12D, Baltimore, MD 21201-1595, USA

## Abstract

Limited, and underutilized, therapeutic options for acute stroke require new approaches to treatment. One such potential approach involves better understanding of innate immune response to brain injury such as acute focal cerebral ischemia. This includes understanding the temporal profile, and specificity, of Toll-like receptor 4 (TLR4) signaling in brain cell types, such as astrocytes, following focal cerebral ischemia. This study evaluated TLR4 signaling, and downstream mediators, in astrocytes, during acute and chronic phases post transient middle cerebral artery occlusion (MCAO). We also determined whether high mobility group box 1 (HMGB1), an endogenous TLR4 ligand, was sufficient to induce TLR4 signaling activation in astrocytes *in vivo and in vitro.* We injected HMGB1 into normal cortex, *in vivo,* and stimulated cultured astrocytes with HMGB1, *in vitro,* and determined TLR4, and downstream mediator, expression by immunohistochemistry. We found that expression of TLR4, and downstream mediators, such as inducible nitric oxide synthase (iNOS), occurs in penumbral astrocytes in acute and chronic phases after focal cerebral ischemia, but was undetectable in cortical astrocytes in the contralateral hemisphere. In addition, cortical injection of recombinant HMGB1 led to a trend towards an almost 2-fold increase in TLR4 expression in astrocytes surrounding the injection site. Consistent with these results, *in vitro* stimulation of the DI TNC1 astrocyte cell line, with recombinant HMGB1, led to increased TLR4 and iNOS message levels. These findings suggest that HMGB1, an endogenous TLR4 ligand, is an important physiological ligand for TLR4 signaling activation, in penumbral astrocytes, following acute and chronic ischemia and HMGB1 amplifies TLR4 signaling in astrocytes.

## 1. Introduction

Stroke is the 5^th^ leading cause of death, and a leading cause of long-term disability, in the U.S [1]. However, there is only one FDA-approved drug for stroke [2] and no drugs that can repair, or improve recovery, once a stroke has occurred [3]. It is well known that the immune response to stroke is robust and continues for up to months following stroke [4]. Therefore, the innate immune response itself remains an attractive target for novel therapeutics that mediate reparative processes and improve recovery. In this regard, the role of innate immune pathways, such as the TLR4 signaling pathway, has been extensively studied in animal models of acute stroke [5–9]. However, TLR4 signaling in astrocytes, a ubiquitous cell type involved in the CNS response to injury, and in reparative processes during the chronic phase of stroke, has not been extensively studied.

Previous studies have shown TLR4 expression in penumbral astrocytes during acute focal cerebral ischemia [5]. More recent studies also show that TLR4 is also expressed by penumbral astrocytes in a model of cortical devascularization [10]. However, it is not known whether TLR4 signaling occurs in these astrocytes during both the acute and chronic phases of focal cerebral ischemia. In addition, it is not known which physiological ligand contributes to such TLR4 signaling, in astrocytes, during focal cerebral ischemia. Previous studies of TLR4 signaling in astrocytes have occurred *in vitro* and focused mostly on lipopolysaccharide (LPS), a ligand for TLR4, which has no physiologic relevance during focal cerebral ischemia [11].

We therefore sought to determine the occurrence of TLR4 expression, as well as that of downstream signaling elements, in the ischemic penumbra, during both the acute and chronic phases of focal cerebral ischemia. We also determined whether HMGB1, a physiologically relevant ligand, known to be released during focal cerebral ischemia [12, 13], contributes to TLR4 signaling in astrocytes in an *in vivo* model of HMGB1 injection into normal rat brain cortex and in cultured astrocytes *in vitro*.

Our results show that TLR4 expression, and downstream signaling activation, occur robustly in penumbral astrocytes during acute focal cerebral ischemia at 48 hrs and during the chronic phase of stroke, up to 7 days after transient MCAO. We also show that injection of HMGB1 into normal rat cortex results in the expression of TLR4, and downstream signaling elements, in perilesional (HMGB1 injection site) astrocytes, compared to injection of vehicle, thereby suggesting that HMGB1 contributes to TLR4 signaling *in vivo* during focal cerebral ischemia. In addition, we show that stimulation of astrocytes, from an astrocyte cell line, with recombinant HMGB1 is sufficient to increase TLR4 mRNA levels *in vitro*.

## 2. Materials and Methods

### 2.1. Transient Middle Cerebral Artery Occlusion (MCAO)

The previously described model of transient MCAO [14] and male wild type Wistar rats (Harlan laboratories, Indianapolis, IN) were employed in these studies. Briefly, following induction of anesthesia, with 60 mg/kg ketamine and 7.5 mg/kg xylazine, intraperitoneally (IP), the skull was thinned 2 mm caudal and 4 mm lateral to the bregma and the Doppler probe attached to the skull with cyanoacrylate glue. Once the internal carotid artery had been identified, it was flushed with a small volume of warm normal saline and the occluder advanced gently, but firmly, under guidance of the Doppler flowmeter (Moor Instruments, Axminster, UK), into the stump of the external carotid artery towards the right MCA. Only animals with greater than 75% drop in relative cerebral blood flow (rCBF) were studied. Animals were maintained under anesthesia and rCBF monitored continuously throughout the duration of the occlusion and for 10 minutes following removal of the occluder.

For acute MCAO, surgery was carried out as above and brains were removed for analysis at 48 hours after MCAO in 5 animals. For chronic MCAO (outside of the 48 hour acute period), surgery was carried out as above, in 3 animals, except that duration of ischemia was 3 hours and a craniectomy was performed at the time of ischemia before removal of the occluder. Brains were removed for analysis 7 days after MCAO surgery.

#### 2.1.1. Cortical HMGB1 Injection

Rats were anesthetized with 60 mg/kg ketamine and 7.5 mg/kg xylazine, and their heads immobilized in a stereotactic frame. Recombinant HMGB1 (rHMGB1) (R&D Systems, Minneapolis, MN), 2.5 *μ*g in 5 *μ*l 1X phosphate buffered saline (PBS) or 5 *μ*l 1X PBS injection was then injected into the overlying cortex using a Hamilton syringe. This dose is in line with HMGB1 doses used in previous HMGB1 injection studies [15]. Three rats each were used for rHMGB1 or PBS injections. Coordinates for cortical injections were at a depth of 2.5 mm from the overlying cortex, 5.5 mm from the midline, and at −4.52 mm bregma. Rats were euthanized 48 hrs following cortical rHMGB1 injection and transcardiac perfusion performed with 4% paraformaldehyde. Brains were placed in 30% sucrose for cryoprotection. Once brains sank to the bottom of the container, 10 *μ*m cryosections were prepared and sections prepared for immunohistochemistry.

#### 2.1.2. Determination of Percentage of TLR4-Positive Astrocytes

We counted the number of TLR4 and GFAP colabeled and total GFAP-positive astrocytes in an equal area adjacent to the peri-injection site. The percentage of TLR4-postive astrocytes was determined as follows: TLR4-GFAP colabeled astrocytes/total number of GFAP-positive astrocytes X 100.

### 2.2. Immunohistochemistry

Following transient MCAO, rats were euthanized at 48 hrs or at 7 days post MCAO and transcardiac perfusion performed with 4% paraformaldehyde. For rHMGB1 studies, animals were euthanized at 48 hrs. Brains from all studies were then prepared for immunohistochemistry, as above, using TLR4-specific antibodies; 1 : 20 (Santa Cruz Biotechnology) and species-specific fluorescently labeled secondary antibodies (Invitrogen) used for detection of TLR4 reactivity in penumbral astrocytes using colabeling following immunoreactivity with anti-GFAP-specific antibodies; 1 : 100 (eBioscience) to identify TLR4 reactivity in penumbral or perilesional astrocytes. Antibodies to known downstream TLR4 mediators such iNOS; at 1 : 400 (Calbiochem) were used to assess downstream TLR4 signaling in penumbral astrocytes.

### 2.3. Real-Time Polymerase Chain Reaction (RT-PCR)

Cells were cultured in Dulbecco's Modified Eagle Media (DMEM) with L-glutamine (Corning) supplemented with 10% HyClone Newborn Calf Serum (Thermo Fisher Scientific) and 1X Pen-strep. Cells were cultured until 80% confluence, and recombinant HMGB1 100 ng/ml (R&D systems) was added to culture as used in previous in vitro stimulation studies [16]. At different time points following HMGB1 stimulation, cells were washed with ice-cold phosphate buffered saline (PBS) (Quality Biologicals Inc. Gaithersburg, MD). Cells were then harvested with a cell scrapper and lysis buffer containing 1% Triton X and protease inhibitor (Roche) in 1X DPBS (Thermo Fisher Scientific). RNA was extracted from cell lysate using Trizol reagent (Thermo Fisher Scientific), according to manufacturer's instructions. To prevent contamination with genomic DNA, RNA was subjected to treatment with Amplification Grade DNase 1 (Invitrogen).

First strand cDNA was prepared from 500 ng of isolated total RNA of each sample, according to manufacturer's instruction, using the First-strand cDNA Synthesis Supermix kit (Invitrogen). Reverse transcription was carried out with Superscript III reverse transcriptase (Invitrogen). For PCR reactions, 1 *μ*l of cDNA reaction and Platinum SYBR Green Super Mix-UDG with ROX (Thermo Fisher Scientific), specific primers, and ultrapure H_2_O were used.

Primers for TLR4 were as follows:

Commercially available Rat QuantiTect primer assay (Qiagen).

Primers for iNOS (Sigma) were
     Forward: CATTCAGATCCCGAAACGCTAC
     Reverse: AGCCTCATGGTGAACACGTTCT
PCR conditions:
     50°C-2 min; 95°C-2 min X1; (95°–15 sec; 60°–30 sec) x 40 cycles and dissociation; 95°–15 sec; 60°-1 min; 95°–15 sec

### 2.4. Statistical Analysis

Where applicable, data are expressed as mean ± standard deviation. Differences between group means were analyzed using the Student's *t* test in Excel. Differences were considered statistically significant when *p* < 0.05. Animals were randomly assigned to groups, and the analysis was performed in a blinded fashion.

## 3. Results

### 3.1. TLR Signaling Occurs in Penumbral Astrocytes during Acute Focal Cerebral Ischemia

**Figure 1:**
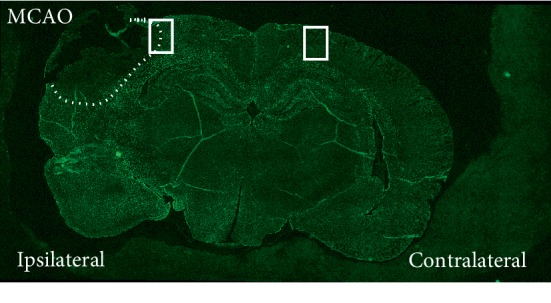
Representative location of GFAP/TLR4 and GFAP/iNOS staining following acute and chronic transient MCAO.

**Figure 2:**
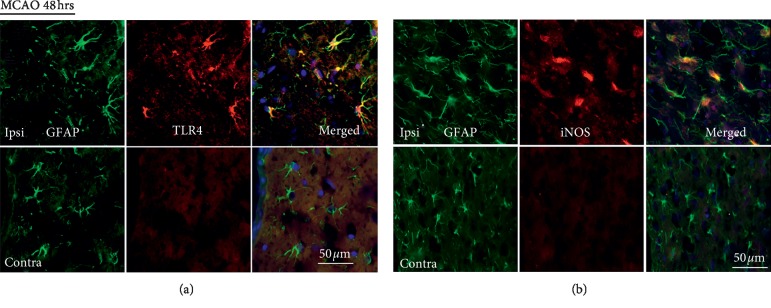
Increased expression of TLR4 and iNOS in penumbral astrocytes during acute focal cerebral ischemia. (a) Upregulation of TLR4 expression in penumbral astrocytes during acute focal cerebral ischemia, 48 hours following MCAO (*n* = 5). Coronal brain sections immunolabeled for GFAP (green) and TLR4 (red), in penumbral astrocytes (merged), in the ipsilateral ischemic hemisphere (upper panel). Brain sections immunolabeled with GFAP (green) and TLR4 (red) in cortical astrocytes (merged) in the contralateral hemisphere. (b) Upregulation of iNOS expression in penumbral astrocytes during acute focal cerebral ischemia (*n* = 5). Brain sections immunolabeled for GFAP (green) and iNOS (red), in penumbral astrocytes (merged), in the ipsilateral ischemic hemisphere (top panel). GFAP expression (green) and iNOS expression (red) in cortical astrocytes (merged) in the contralateral hemisphere (lower panel). Scale bar = 50 *μ*m.

Astrocytes, located in the penumbra (Figure 1), express TLR4 protein, and downstream mediators such as iNOS, 48 hrs following focal cerebral ischemia. In addition, of note, expression of TLR4 and its downstream mediators occurs in hypertrophic and reactive penumbral astrocytes as determined by concomitant increased GFAP expression. In contrast, there is no discernible TLR4, or downstream mediator protein expression, in GFAP-positive cortical astrocytes in the contralateral nonischemic hemisphere; Figure 2.

#### 3.1.1. TLR Signaling in Penumbral Astrocytes during Chronic Focal Cerebral Ischemia

**Figure 3:**
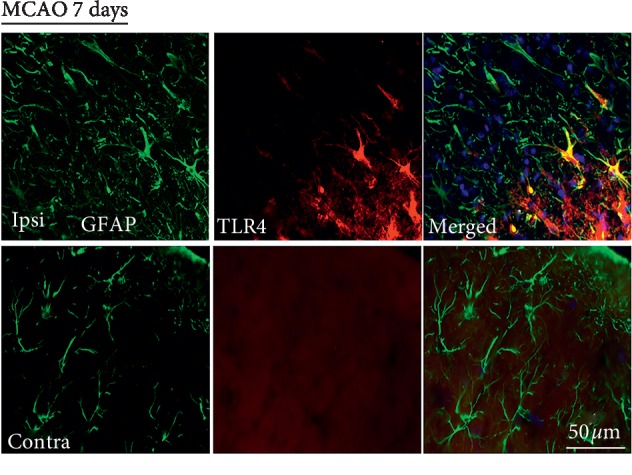
TLR4 expression is increased in penumbral astrocytes during chronic focal cerebral ischemia, 7 days post 3 h MCAO (*n* = 3). Brain sections immunolabeled for GFAP (green) and TLR4 (red), in penumbral astrocytes (merged), in the ipsilateral ischemic hemisphere (upper). Brain sections immunolabeled for GFAP (green) and TLR4 (red) in cortical astrocytes (merged) in the contralateral hemisphere (lower panel). Scale bar = 50 *μ*m.

Penumbral astrocytes also express TLR4 during chronic focal cerebral ischemia. These TLR4-expressing astrocytes are reactive, hypertrophic, and GFAP-positive. In contrast, there is no detectableTLR4 expression in GFAP-positive cortical astrocytes on the contralateral nonischemic hemisphere; Figure 3.

### 3.2. HMGB1 Increases TLR4 Signaling in Cortical Astrocytes

**Figure 4:**
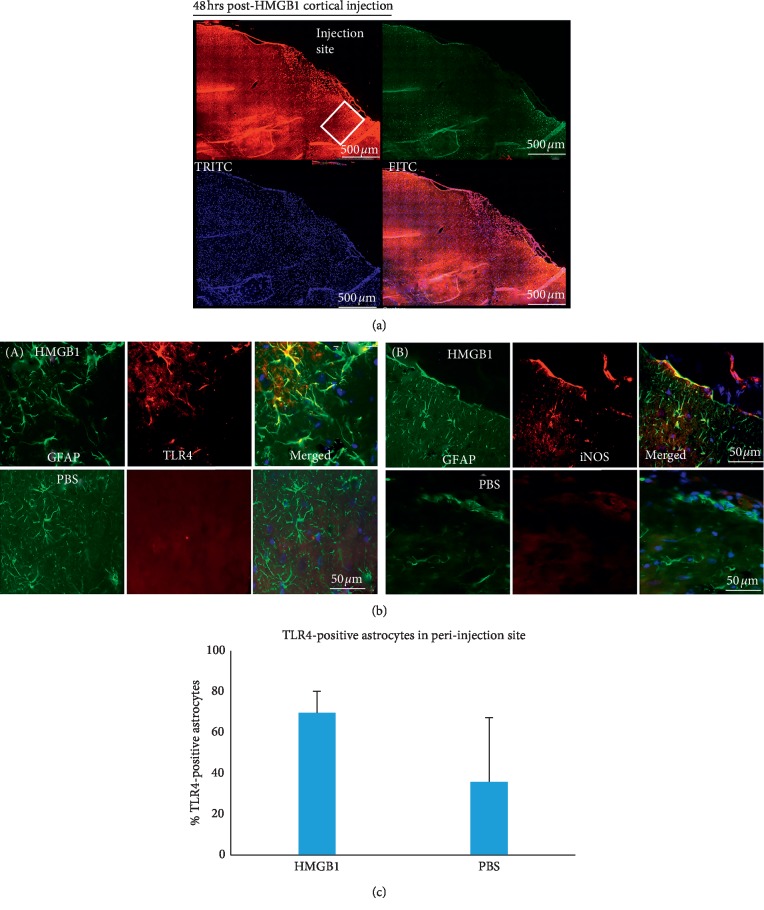
(a) Representative area of GFAP/TLR4 and iNOS/TLR4 staining following HMGB1 cortical injection. (b) Increase in TLR4 and iNOS expression in astrocytes following cortical injection of HMGB1 (*n* = 3). (A) Upregulation of TLR4 expression in perilesional astrocytes 48 hours following injection of recombinant HMGB1 into normal rat cortex. Immunofluorescence staining of representative 10 *μ*m coronal brain sections; GFAP (green) expression and TLR4 (red) expression in perilesional astrocytes (merged) (upper panel). TLR4 expression in perilesional astrocytes following injection of PBS into normal rat cortex. GFAP (green) expression and TLR4 (red) expression in perilesional astrocytes (merged) in the contralateral hemisphere (lower panel). (B) Upregulation of iNOS expression in perilesional astrocytes 48 hours following injection of recombinant HMGB1 into normal rat cortex. Immunofluorescence staining of representative 10 *μ*m coronal brain sections; GFAP (green) expression and iNOS (red) expression, in perilesional astrocytes (merged) (upper panel). iNOS expression in perilesional astrocytes following injection of phosphate buffered saline into normal rat cortex. GFAP expression (green) and TLR4 expression (red) in perilesional astrocytes (merged) in the contralateral hemisphere (lower panel). Brain sections are representative sections from 3 animals each injected with HMGB1 and 3 animals injected with PBS. Scale bar = 50 *μ*m. (c) Percentage of TLR4-postitive astrocytes in the peri-injection site following injection of rHMGB1 into normal rat cortex. There is a trend towards an almost 2-fold increase in the percentage of TLR4-positive astrocytes in the peri-injection site following injection of rHGMB1 compared to injection of PBS; 69.7 ± 10.5% compared to 35.9 ± 31.4%; *p*=0.1527 (*n* = 3 each per group for rHMGB1 injection and PBS injection).

Injection of recombinant HMGB1 (Figure 4(a)), a known endogenous TLR4 ligand released during focal cerebral ischemia, results in a trend towards an almost 2-fold increase in expression of TLR4, when injected into normal rat cortex; Figure 4(b) ((A) and (C)). In addition, expression of iNOS, a downstream mediator of TLR4, appears increased following injection of rHMGB1 compared to following injection of PBS; Figure 4(b) (B). Specifically, injection of rHMGB1 into normal rat cortex resulted in 69.7 ± 10.5% TLR4-positive astrocytes compared to 35.9 ± 31.4% TLR4-positive astrocytes following injection of PBS; *p*=0.1527 (*n* = 3 per group) (Figure 4(c)).

### 3.3. In Vitro Stimulation of Astrocytes, with HMGB1, Increases TLR4 and Downstream Mediator Message Levels

**Figure 5:**
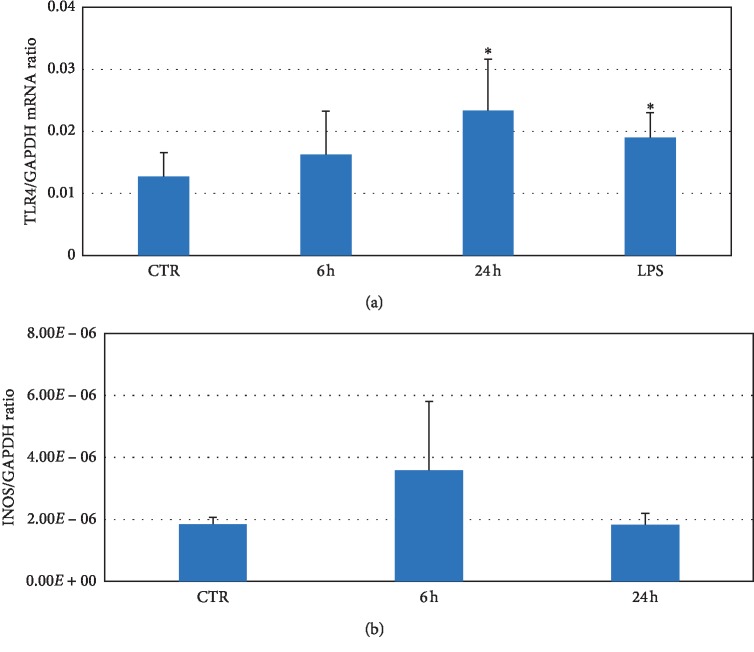
Increase in TLR4 signaling in cultured astrocytes following stimulation with recombinant HMGB1 (*n* = 4 independent experiments). (a) Significant increase in TLR4 message levels in DI TNC1 cells stimulated with recombinant HMGB1 compared to cells stimulated with PBS, using GADPH as loading control and housekeeping gene. (b) Increase in iNOS message levels in DI TNC1 cells stimulated with recombinant HMGB1 compared to cells stimulated with PBS, using GADPH as loading control and housekeeping gene. These data are average from 3 independent experiments; ^*∗*^=*p* < 0.05.

Stimulation of the astrocyte cell line, DI TNC1 (American Type Culture Collection), with 100 ng/ml of recombinant HMGB1 for 24 hrs leads to significant increase in TLR4 mRNA levels by RT-PCR compared to PBS-stimulated cells. Stimulation of cells with LPS, as positive control, also leads to a significant increase in TLR4; Figure 5(a). In addition, stimulation of the same astrocyte cell line, DI TNC1 in a similar manner, with recombinant HMGB1 leads to increase in iNOS message levels, compared to PBS stimulated cells, using expression of housekeeping gene GAPDH expression as control; Figure 5(b).

## 4. Discussion

These studies show the temporal profile and cell specificity of TLR4 signaling in focal cerebral ischemia. We show TLR4 signaling in astrocytes, an important and ubiquitous central nervous system (CNS) cell type, which is important in maintaining brain homeostasis and the innate response to brain injury. Specifically, we show that TLR4 expression and signaling occurs not only in penumbral astrocytes in acute focal cerebral ischemia, but also in chronic focal cerebral ischemia. In addition, we show that HMGB1, an important endogenous TLR4 ligand, increases TLR4 expression, and signaling, in cortical astrocytes in an injection model. Consistent with these findings, we demonstrate that *in vitro* stimulation of cultured astrocytes with recombinant HMGB1 leads to increased TLR4 and downstream mediator expression.

Our current results showing TLR4 signaling in penumbral astrocytes, in both acute and chronic focal cerebral ischemia, suggest a possible biphasic role for TLR4 signaling in cerebral ischemia. These findings are consistent with previous studies showing biphasic actions of HMGB1, a TLR4 ligand, in focal cerebral ischemia [17, 18]. In previous studies, some of the biphasic actions of HMGB1 were attributed to release of HMGB1 from activated astrocytes stimulated with IL-1*β* [19]. Our current results showing that HMGB1 stimulation of astrocytes increases TLR4 expression in cortical astrocytes, *in vivo* and *in vitro,* are compatible with a paradigm in which autocrine TLR4 signaling, via HMGB1, leads to astrocyte activation during chronic cerebral ischemia Figure 5. On the other hand, release of HMGB1 from dying neurons, in the acute phase, most likely plays an important role in astrocyte activation early during focal cerebral ischemia, possibly in a paracrine fashion. In addition to dying neurons, microglia can also be a source of HMGB1 [20, 21]. This proposed model of HMGB1/TLR4 signaling, in focal cerebral ischemia, is consistent with previous studies in which autocrine/paracrine HMGB1 signaling occurs in response to hypoxia in smooth muscle cells [22].

The detrimental role of innate immune signaling, such as TLR4 signaling, in acute experimental focal cerebral ischemia has been well established in studies employing TLR4 antagonist, TAK-242 [23, 24]. In contrast, it is not known what the role of TLR4 signaling, and specifically, astrocyte TLR4 signaling, is in chronic cerebral ischemia. Therefore, future studies will determine the role of TLR4 signaling in repair and recovery in focal cerebral ischemia. To this effect, models of spinal cord injury suggest a role for TLR4 signaling in repair and recovery [25, 26]. A role for the TLR4 signaling pathway in repair and recovery is further suggested by studies that implicate TLR4 in wound healing in other organ systems [27]. With regards to astrocyte activation, recent studies show that reactive astrocytes consist of A1 and A2 astrocytes that are induced by M1 or M2 microglia, respectively [28]. Of note, these A1 and A2 astrocytes were induced by treatment with another known TLR4 agonist, LPS. Future studies will therefore explore the role of HMGB1/TLR4 astrocyte signaling in the induction of A2 reactive astrocytes that express neurotrophic factors known to be involved in recovery and repair [28].

Our current results showing that stimulation of cultured astrocytes from an astrocyte cell line, with lipopolysaccharide (LPS), another TLR4 ligand, increases TLR4 message levels in astrocytes concurs with previous studies also showing that LPS increases TLR4 levels in astrocytes [29]. It is important to note that we did not observe TLR4 *protein* staining in the contralateral nonischemic hemispheres in our acute and chronic ischemia models. These studies thereby suggest that TLR4 protein is not expressed in detectable amounts, using immunohistochemistry, under basal conditions in normal tissue. However, during injury, there appears to be at least a small amount of TLR4 able to initiate ligand binding of various danger-associated molecular patterns (DAMPs) and subsequent signal amplification [10, 30]. However, we did observe baseline TLR4 *message* levels in our cultured astrocyte cell line stimulated with PBS; Figure 5(a). We also observed expression of TLR4 protein in unstimulated DI-TNC1 cells; Supplementary data; [Supplementary-material supplementary-material-1]. Phospho P65 staining also colocalized with TLR4 staining in these unstimulated cells; [Supplementary-material supplementary-material-1]. The presence of baseline TLR4 levels in astrocytes is consistent with previous studies showing the presence of TLR4 mRNA in differentiated astrocytes [31].

**Figure 6:**
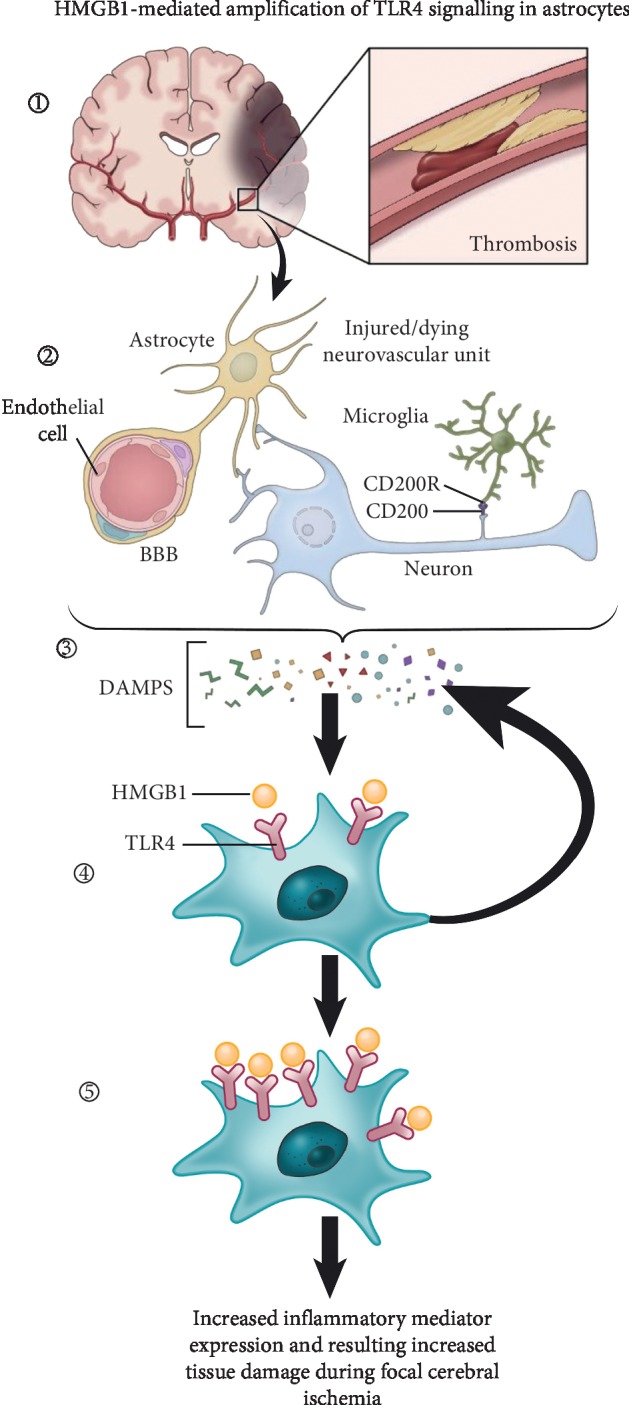
HMGB1-mediated amplification of TLR4 signaling in astrocytes. Schematic showing HMGB1-mediated increase in astrocyte TLR4 expression. Release of danger-associated molecular patterns, including HMGB1 from dead or dying components of the neurovascular unit, following focal cerebral ischemia leads to increased astrocyte TLR4 expression. HMGB1-mediated astrocyte signaling is a potential method of amplification of tissue damage in focal cerebral ischemia.

In addition, in support of our current results are reports showing that the HMGB1/TLR4 signaling pathway has been shown to play an important role in astrocyte activation in other neurological conditions such as seizures [32], further highlighting the importance of this pathway in astrocytes as a potential mechanism of amplification of tissue damage during focal cerebral ischemia (Figure 6).

Some of the limitations of our current study include the fact that HMGB1 binds to other receptors other than TLR4 such as TLR2 and RAGE [17, 33]. However, our current studies focus on the HMGB1/TLR4 signaling pathway because previous studies have shown that TLR4 receptors outnumber TLR2 receptors in astrocytes [16]. In addition, the TLR4 signaling pathway has been studied more extensively in focal cerebral ischemia [5, 7–9].

Lastly, while several downstream inflammatory mediators can be used as a surrogate for TLR4 signaling, we focused on iNOS as a downstream mediator of astrocyte TLR4 signaling because iNOS is a reliable indicator of astrocyte activation [34]. In addition, previous studies also show that iNOS expression is decreased in TLR4-deficient mice [5].

## 5. Conclusions

These studies show that HMGB1, a physiologically relevant endogenous TLR4 ligand released during focal cerebral ischemia, is sufficient to induce TLR4 expression in astrocytes both *in vivo* and *in vitro*. These findings suggest a role for a novel, endogenous paracrine and autocrine HMGB1/TLR4 signaling pathways in astrocytes during acute and chronic and focal cerebral ischemia, respectively. Further studies are needed to determine the role of TLR4 signaling in penumbral astrocytes during chronic focal ischemia and the role of HMGB1-TLR4 signaling in activation of A1 vs A2 reactive astrocytes.

## Data Availability

Raw data supporting the results reported in this article are available upon reasonable request by contacting the corresponding author BMF. The University of Maryland, Baltimore, does not participate in a formal mechanism for anonymous sharing of data.

## Supplementary Materials

Supplementary Materials Materials and methods: DI-TNC1 cell culture and immunostaining: Cell culture: DI-TNC1 cells (ATCC) were grown in Dulbecco's Modified Eagle's Medium (DMEM) (ATCC) containing 10% fetal bovine serum (FBS) and 1% Pen-strep; (both from ATCC), on poly L-lysine-coated glass cover slides (Corning) in 12-well plates at a concentration of 0.8X105/ml. Cells were maintained at 37°C in an incubator humidified with 5% CO_2_. Immunostaining: after culturing cells overnight to 48 hours, cells were washed twice with ice-cold PBS and fixed with fixation buffer (BD Biosciences) for 20 minutes, at room temperature, and rinsed three times with PBS. Cells were then permeabilized with 0.1% Triton X, in 1X PBS for 20 minutes, at room temperature, and washed twice with PBS. Nonspecific binding was blocked with 2% donkey blocker for 1 hour at room temperature. Subsequently, cells were incubated overnight with primary antibodies: Anti-TLR4: 1 : 500 (Invitrogen, #48-2300), Anti-GFAP; 1:100 (Invitrogen, #14-9892-82), and Anti-phospho P-65; 1:100 (Cell Signaling Technology, # 3036S). After washing three times with PBS, cells were incubated with secondary antibodies (donkey anti-mouse: 1 : 500 (#A31570), donkey anti-rabbit: 1 : 500 (#A21206); both Invitrogen) for one hour at room temperature. Following secondary antibody incubation, cells were rinsed with PBS and counterstained with DAPI Antifade (Life Technologies) and imaged under a fluorescent microscope. Results: DITNC1 cells express basal levels of TLR4. Following immunohistochemistry with anti-TLR4 and anti-GFAP antibodies, DI‐TNC‐1 cells showed basal expression of surface TLR4 that colocalized with GFAP staining in merged images; Figure S1; (*n* = 2). DITNC1 cells express basal levels of phospho‐p65. Following immunohistochemistry with anti‐phospho‐p65 and anti-GFAP antibodies, DI-TNC-1 cells showed basal expression of phospho‐p65 in the nucleus that colocalised with GFAP staining; Figure S2; (*n* = 2).
