# Supplementary material for: HMGB1 is a Potential Mediator of Astrocytic TLR4 Signaling Activation following Acute and Chronic Focal Cerebral Ischemia
Source: Neurol Res Int. 2020 Feb 20;2020:3929438. doi: 10.1155/2020/3929438 (PMC7053497; doi:10.1155/2020/3929438)
Supplement: Supplementary Materials — Materials and methods: DI-TNC1 cell culture and immunostaining: Cell culture: DI-TNC1 cells (ATCC) were grown in Dulbecco's Modified Eagle's Medium (DMEM) (ATCC) containing 10% fetal bovine serum (FBS) and 1% Pen-strep; (both from ATCC), on poly L-lysine-coated glass cover slides (Corning) in 12-well plates at a concentration of 0.8X105/ml. Cells were maintained at 37°C in an incubator humidified with 5% CO2. Immunostaining: after culturing cells overnight to 48 hours, cells were washed twice with ice-cold PBS and fixed with fixation buffer (BD Biosciences) for 20 minutes, at room temperature, and rinsed three times with PBS. Cells were then permeabilized with 0.1% Triton X, in 1X PBS for 20 minutes, at room temperature, and washed twice with PBS. Nonspecific binding was blocked with 2% donkey blocker for 1 hour at room temperature. Subsequently, cells were incubated overnight with primary antibodies: Anti-TLR4: 1 : 500 (Invitrogen, #48-2300), Anti-GFAP; 1:100 (Invitrogen, #14-9892-82), and Anti-phospho P-65; 1:100 (Cell Signaling Technology, # 3036S). After washing three times with PBS, cells were incubated with secondary antibodies (donkey anti-mouse: 1 : 500 (#A31570), donkey anti-rabbit: 1 : 500 (#A21206); both Invitrogen) for one hour at room temperature. Following secondary antibody incubation, cells were rinsed with PBS and counterstained with DAPI Antifade (Life Technologies) and imaged under a fluorescent microscope. Results: DITNC1 cells express basal levels of TLR4. Following immunohistochemistry with anti-TLR4 and anti-GFAP antibodies, DI‐TNC‐1 cells showed basal expression of surface TLR4 that colocalized with GFAP staining in merged images; Figure S1; (n = 2). DITNC1 cells express basal levels of phospho‐p65. Following immunohistochemistry with anti‐phospho‐p65 and anti-GFAP antibodies, DI-TNC-1 cells showed basal expression of phospho‐p65 in the nucleus that colocalised with GFAP staining; Figure S2; (n = 2). [file 3929438.f1.zip › Supplementary materials and methods -Neurology Research International Dec 2019.docx]

**Supplementary data:**

**Materials and methods:**

**DI-TNC1 cell culture and immunostaining:**

**Cell culture:**

DI-TNC1 cells (ATCC) were grown in Dulbecco’s Modified Eagle’s Medium (DMEM) (ATCC) containing 10% Fetal Bovine Serum (FBS) and 1% Pen-strep; (both from ATCC), on Poly L-lysine coated glass cover slides (Corning) in 12-well plates at a concentration of 0.8X10^5^/ml. Cells were maintained at 37^O^C in an incubator humidified with 5% CO2.

**Immunostaining:**

After culturing cells overnight to 48 hours, cells were washed twice with ice-cold PBS and fixed with fixation buffer (BD Biosciences) for 20 minutes, at room temperature, and rinsed three times with PBS. Cells were then permeabilized with 0.1% Triton X, in 1XPBS for 20 minutes, at room temperature, and washed twice with PBS. Non-specific binding was blocked with 2% donkey blocker for 1 hour at room temperature. Subsequently, cells were incubated overnight with primary antibodies; Anti-TLR4: 1: 500 (Invitrogen, #48-2300), Anti-GFAP; 1:100 (Invitrogen, #14-9892-82), Anti-phospho P-65; 1:100 (Cell Signaling Technology, # 3036S). After washing three times with PBS cells were incubated with secondary antibodies; (donkey anti-mouse: 1:500 (#A31570), donkey anti- rabbit: 1:500 (#A21206); both Invitrogen) for one hour at room temperature. Following secondary antibody incubation, cells were rinsed with PBS and counterstained with DAPI Anti fade (Life technologies) and imaged under a fluorescent microscope.

**Results:**

**DI-TNC-1 cells express basal levels of TLR4.**

Following immunohistochemistry with anti-TLR4 and anti-GFAP antibodies, DI-TNC-1 cells showed basal expression of surface TLR4 which colocalized with GFAP staining in merged images; Figure S1; (n=2).

**DI-TNC-1 cells express basal levels of p65**

Following immunohistochemistry with anti-p65 and anti-GFAP antibodies, DI-TNC-1 cells showed basal expression of p65 in the nucleus which colocalised with GFAP staining; Figure S2; (n=2).
